# A Spatiotemporal Feature-Based Approach for the Detection of Unlicensed Taxis in Urban Areas

**DOI:** 10.3390/s24248206

**Published:** 2024-12-23

**Authors:** Yun Xiao, Rongqiao Li, Jinyan Li

**Affiliations:** 1 School of Urban Construction and Transportation, Hefei University, Hefei 230601, China; xiaoyun@hfuu.edu.cn (Y.X.); jiny1107@126.com (J.L.); 2 School of Civil Engineering and Transportation, Northeast Forestry University, Harbin 150040, China

**Keywords:** traffic engineering, unlicensed taxis, binary logistic regression, spatiotemporal feature mining

## Abstract

Unlicensed taxis seriously disrupt the transportation market order, and threaten passenger safety. Therefore, this paper proposes a method for identifying unlicensed taxis based on travel characteristics. First, the vehicle mileage and operation time are calculated using traffic surveillance bayonet data, and variance analysis is applied to identification indicators for unlicensed taxis. Secondly, the mathematical model for identifying unlicensed taxis is established. The model is validated using the Hosmer–Lemeshow test, confusion matrix and ROC curve analysis. Finally, by applying methods such as geographic information matching, the spatiotemporal distribution characteristics of suspected unlicensed taxis in a city in Anhui Province are identified. The results show that the model effectively identifies suspected unlicensed taxis (ACC = 99.10%). The daily average mileage, daily average operating time, and number of operating days for suspected unlicensed taxis are significantly higher than those for private cars. Additionally, the suspected unlicensed taxis exhibit regular patterns in their travel origin–destination points and temporal distribution, enabling traffic management authorities to implement targeted regulatory measures.

## 1. Introduction

Unlicensed taxis refers to vehicles engaged in transportation operations without legally obtaining the necessary operating rights, posing significant safety risks to the transportation sector [1]. Currently, many drivers in China are involved in unlicensed taxi operations. Due to the lack of necessary assessments and qualification checks, the quality of drivers is inconsistent. Some even have histories of criminal records, posing significant threats to passenger safety. Additionally, most unlicensed taxis are low-end or poorly maintained vehicles, making it difficult to ensure vehicle quality, which poses potential risks to passenger safety. Traditional methods for identifying unlicensed taxis primarily rely on manual roadside inspections and passenger reports, which not only consume substantial human and material resources but also easily disrupt normal traffic order, leading to social conflicts. Therefore, enhancing traditional methods by leveraging traffic big data analysis to identify unlicensed taxi operations is crucial for optimizing road transportation management.

Traffic surveillance bayonet data contain a vast amount of vehicle travel information, from which vehicle travel characteristics can be effectively extracted, enabling accurate identification of unlicensed vehicles and providing a new approach to managing these vehicles. Some scholars have utilized big data mining techniques to extract vehicle travel information from traffic surveillance bayonet data. Liu et al. [2] analyzed vehicle travel characteristics based on frequent sequence-pattern mining algorithms using bayonet vehicle trajectory sequences. Wang et al. [3] collected hourly scale data on the structure of vehicle types, road types, and time-varying characteristics of travel and technical parameters for non-local vehicles in the road network inside and outside the First Ring of Foshan City using big data mining techniques. Chen et al. [4] proposed a method for completing vehicle travel OD based on license plate recognition data, supplementing and restoring OD at measurement points by distributing link flows to obtain complete vehicle travel paths. Wei et al. [5] established a vehicle travel-demand evaluation index system based on traffic surveillance bayonet data and analyzed vehicle travel characteristics from multiple dimensions. Long et al. [6] extracted vehicle travel-path information from traffic surveillance bayonet data and analyzed vehicle travel patterns at both individual and aggregate levels. Ruan et al. [7] combined the K-shortest path algorithm with the grey relational algorithm to complete decisions and reconstruct travel paths generated from license-plate recognition data. Yao et al. [8] used bayonet license-plate recognition data and employed factor analysis to integrate vehicle travel stability factors, efficiently identifying commuter vehicles. Wu et al. [9] introduced a heuristic optimal scheduling approach to identify abnormal traffic events, and the results demonstrated that this method outperforms existing algorithms. The above studies are based on traffic surveillance bayonet data, and set feature indicators according to vehicle travel patterns to extract commuter vehicle travel characteristics, providing a methodological reference for extracting and identifying the features of suspected unlicensed taxis.

Currently, scholars both domestically and internationally have conducted extensive research on identifying unlicensed taxis. Lin et al. [10] proposed a reporting system for unlicensed vehicles based on Near Field Communication technology and Android, encouraging passengers to report suspected unlicensed taxis and creating an effective environment for their governance. Wang et al. [11] used Convolutional Neural Networks (CNNs) to obtain trajectory data of unlicensed and normal vehicles through simulation experiments, performing feature learning and recognition to improve the identification rate of suspected unlicensed taxis. Li et al. [12] used vehicle mileage as an identification indicator to establish a model for identifying unlicensed taxis and identifying suspected unlicensed taxis through case analysis. Shuai et al. [13] extracted vehicle Radio Frequency Identification data and proposed a k-medoids-based algorithm for identifying unlicensed taxis, which was validated through experiments. Ma et al. [14] collected vehicle operation data using RFID technology and applied the SOM neural network clustering algorithm to establish a mathematical model for identifying unlicensed taxis, achieving favorable results. Tian et al. [15] developed a method for identifying unlicensed taxis based on ETC toll data, employed an improved K-means++ algorithm, and empirically identified unlicensed taxis in Guiyang. The focus of the above studies is on algorithm models, but they primarily rely on data simulation for theoretical validation, and their applicability requires further testing.

With the development of big-data analysis techniques, some scholars have conducted research on identifying unlicensed taxis by analyzing various types of traffic data and extracting different feature indicators. Zhao et al. [16] analyzed vehicle refueling data, extracting temporal and spatial refueling characteristics, and defined vehicles with abnormal refueling patterns as suspected unlicensed taxis. Chen et al. [17] used Electronic Registration Identification data to build a detection model for unlicensed taxis using ensemble learning methods, identifying suspected unlicensed taxis. Yuan et al. [18] extracted vehicle travel data from traffic surveillance bayonets, developed a detection model to identify coarse-grained unlicensed taxis and further applied a feature-trained Support Vector Machine classification model to identify fine-grained unlicensed taxis. Wang et al. [19] extracted two types of behavioral features: daily behaviors and sustainable behaviors, and used three machine learning methods to compare the accuracy and quantity of identified unlicensed taxis by each method. Tian et al. [20] proposed two indicators: “path irregularity” and “time irregularity”, and developed a model to distinguish between commercial and non-commercial vehicles. Huang et al. [21] employed a random forest algorithm to develop a classifier for identifying unlicensed taxis, comparing its performance with that of other models, to validate the accuracy of the proposed method. Juan et al. [22] utilized mobile GPS data to develop a decision-tree machine-learning classification algorithm, which can be applied to urban traffic monitoring. The above studies used machine learning algorithms to identify suspected unlicensed taxis in the study areas, but they did not further analyze the spatiotemporal trajectory characteristics of these vehicles, limiting their ability to provide strong support for precise management by authorities.

Therefore, this study extended previous research by using traffic surveillance bayonet data to gather vehicle passage information. The spatiotemporal characteristic indicators of private cars, confirmed unlicensed taxis and compliant taxis were selected and analyzed using variance analysis to explore their differences, thereby determining the identification indicators for unlicensed taxis. Based on this, a Binary Logistic Regression analysis was conducted to develop a model for identifying unlicensed taxis using significant identification indicators. The high-precision spatiotemporal characteristics of suspected unlicensed taxis were then extracted, and governance measures for these vehicles were proposed. The research results contribute to enriching the methods for identifying and regulating unlicensed taxis, providing strategic guidance for traffic management authorities in formulating policies to combat illegal operations, thereby enhancing law enforcement efficiency and passage safety. The primary contributions of this study are summarized as follows:

(1) This study addresses the limitations of previous research, which was constrained by a narrow range of data types and small sample sizes. Unlike prior studies that mined data from the vehicle’s perspective, this study adopts the perspective of traffic managers, utilizing traffic surveillance bayonets distributed across the road network to collect vehicle passage data. This method facilitates the convenient and accurate gathering of multidimensional traffic data from vehicles on the road, providing a robust data foundation for identifying unlicensed taxis and ensuring the successful implementation of practical applications.
(2) This study avoided relying on a single vehicle characteristic by incorporating multiple features, such as daily average mileage, daily operating time, and the ratio of operating days. Through multidimensional cross-analysis, this approach improved identification accuracy and bolstered the model’s robustness, mitigating biases associated with relying on a single indicator.
(3) To develop effective solutions for the precise regulation of unlicensed taxis, this study analyzed the spatiotemporal distribution of suspected unlicensed taxis, including their operating start- and end-points and times, to identify distribution patterns. This thorough analysis provides a strong basis for traffic management authorities to implement targeted regulations, thereby improving management efficiency.

## 2. Multi-Source Data Processing

### 2.1. Data Collection and Filtering

#### 2.1.1. Data Collection

Traffic surveillance bayonet data are characterized by strong timeliness and high quality, accurately reflecting traffic flow and vehicle trajectories. This is one of the essential foundational datasets for traffic big data analysis [23,24,25]. Thees data include vehicle passage data and bayonet location information, with their types and meanings shown in Table 1 and Table 2.

This study collected 42.045 million vehicle passage records over ten days in a city in Anhui Province through traffic surveillance bayonets. Irrelevant data attributes were removed according to the research objectives, and each vehicle passage record *r* can be represented by Equation (1).

(1) $$r_{i}=(vnum_{i},vncol_{i},tscnum_{i},ptime_{i},pspeed_{i})$$

where *i* represents the *i*-th vehicle passage data recorded by the traffic surveillance bayonet.

By sorting each vehicle’s passage records in chronological order based on the time they passed through the traffic surveillance bayonets, the resulting set of passage records for each vehicle, denoted as *R*, can be represented by Equation (2).

(2) $$R_{j}=\left\{r_{1},r_{2},\cdots ,r_{i}\right\}$$

where *R_j_* represents the set of passage records for vehicle *j*, ordered by time.

#### 2.1.2. Data Filtering

When collecting vehicle passage data through traffic surveillance bayonets, irrelevant special vehicle passage data may be recorded, and noise data may be generated due to factors such as weather and equipment conditions [26,27]. To improve the accuracy of vehicle travel-information estimation, the raw bayonet data need to be processed as follows:

(1) Step 1: Exclude Special Vehicle Data

Special vehicles include trucks, police cars, government vehicles, driving test vehicles, taxis, etc., which are unrelated to unlicensed taxi operations. This study filters out special vehicle data based on the vehicle numbering rules in China and the vehicle information provided by the relevant authorities.

(2) Step 2: Eliminating Noisy Data

When calculating various vehicle indicators based on the data collected from traffic surveillance bayonets, noise data can increase calculation errors and even produce incorrect results. Common types of noise data include missing data, duplicate data, and erroneous data. The types of noise data generated by the traffic surveillance bayonet in this city are shown in Table 3. This study addresses missing data according to the following steps.

(a) Retrieval of Noise Data

Based on the vehicle passage records processed in Step 1, a binary function *ψ* is constructed to determine whether the *vnum_i_* data in *r_i_* is abnormal, as shown in Equation (3).

(3) $$\psi_{i}=\left\{\begin{array}{c} 0,\;abnormal\;vehicle\;license\;plate\\ 1,\;normal\;vehicle\;license\;plate \end{array}\right.$$

(b) Similarity Analysis Between Noise Data and Normal Data

Common methods for similarity analysis between noise data and normal data include Cosine Similarity, Jaccard Similarity, and Levenshtein Distance. Due to its intuitiveness, simplicity, and sensitivity to editing operations, this study uses Levenshtein Distance for the similarity analysis between noise data and normal data. Levenshtein Distance refers to the minimum number of editing operations required to transform string *S* into target string *T*. The editing operations include insertion, deletion, and substitution of individual characters. The calculation formula is shown in Equation (4) [28].

(4) $$lev_{S,T}(i,j)=\left\{\begin{array}{c} \max(i,j)\\ \min\left\{\begin{array}{c} lev_{S,T}(i-1,j)+1\\ lev_{S,T}(i,j-1)+1\\ lev_{S,T}(i-1,j-1)+1_{\left(S_{i}\neq T_{j}\right)} \end{array}\right. \end{array}\right.\begin{array}{c} \begin{array}{cc} if & \min(i,j)=0 \end{array}\\ otherwise \end{array}$$

where *S_i_* and *T_j_* represent the *i*-th and *j*-th characters of strings *S* and *T*, respectively; 1(*S_i_* ≠ *T_j_*) equals 1 when *S_i_* ≠ *T_j_*, otherwise it equals 0.

If *S* and *T* are two different strings, their similarity can be calculated using Equation (5).

(5) $$\gamma =1-\frac{lev_{S,T}(i,j)}{\max(L_{S},L_{T})}$$

where *L_S_* and *L_T_* represent the lengths of strings *S* and *T*, respectively. The larger the value of *γ*, the higher the similarity between *S* and *T*.

The differentiation between the two sets of license plate data, *ψ* = 0 and *ψ* = 1, was quantitatively assessed using the Levenshtein Distance, resulting in the similarity *γ* between abnormal and normal license plates.

(c) Data Supplementation and Removal

For each abnormal license plate and normal license plate, a similarity comparison is performed. If the abnormal license plate *vnum_i_* has the highest similarity with the normal license plate *vnum_j_*, and the passage records of *R_i_* and *R_j_* show temporal continuity, then *R_i_* is merged into *R_j_* for data supplementation. Data that do not meet these conditions are considered invalid and are removed.

(3) Step 3: Invalid Data Filtering

The distance Δ*S* between the traffic surveillance bayonets corresponding to two consecutive passage records of vehicle *j*, as well as the time difference Δ*t* between these records, was calculated. The spatial movement speed *v*_0_ = Δ*S*/Δ*t* of vehicle *j* within the adjacent traffic surveillance bayonets is then determined. If *v*_0_ > 100 km/h, the passage record is marked as invalid and is removed.

### 2.2. Training Sample Selection

Unlicensed taxis and compliant taxis (including traditional taxis and ride-hailing vehicles) have similar operating patterns, which differ to some extent from those of regular private cars [17]. Analyzing the similarities and differences between them can effectively capture their operational characteristics. Therefore, this study extracts historical driving data from a subset of private cars, confirmed unlicensed taxis, and compliant taxis as the training sample *υ*, used for identifying indicators and analyzing operational characteristics. The expression is given in Equation (6). The training sample *υ* consists of the set of passage records from private cars *υ_N_*, confirmed unlicensed taxis *υ_I_*, and compliant taxis *υ_L_*.

(6) $$\left\{\begin{array}{c} \upsilon_{N}=\left\{R_{1},R_{2},\cdots ,R_{n}\right\}\\ \upsilon_{I}=\left\{R_{1},R_{2},\cdots ,R_{m}\right\}\\ \upsilon_{L}=\left\{R_{1},R_{2},\cdots ,R_{g}\right\} \end{array}\right.$$

where *n*, *m*, and *g* represent the number of private cars, confirmed unlicensed taxis, and compliant taxis, respectively.

Based on the bayonet data from 10 days, this study selected the passage records of 1852 compliant taxis, 121 confirmed unlicensed taxis, and a random sample of 2000 private cars in the city as the training set for further analysis.

## 3. Identification Indicators

### 3.1. Operational Characteristic Indicators of Unlicensed Taxis

Unlicensed taxis typically exhibit the following characteristics: (1) lower vehicle prices; (2) higher maximum passenger capacity; (3) longer driving times; and (4) greater driving distances [16]. Based on this, the definitions of the operational characteristic indicators for unlicensed taxis are provided.

(1) Definition 1: Average Daily Mileage

The average daily mileage (*Sa*) is defined as the ratio of the total mileage *S_i_* of vehicle *i* during a statistical period of *T* days to the number of days *d_i_* the vehicle was in operation, as shown in Equation (7).

(7) $$Sa_{i}=\frac{S_{i}}{d_{i}}=\frac{\sum_{j=1}^{T}s_{i,j}}{d_{i}}$$

where *s_i,j_* represents the mileage of vehicle *i* on the *j*-th day during the statistical period *T*, measured in kilometers.

(2) Definition 2: Average Daily Operating Time

The average daily operating time (*Ta*) is defined as the ratio of the total operating time *To_i_* of vehicle *i* during a statistical period of *T* days to the number of days *d_i_* the vehicle was in operation, as shown in Equation (8).

(8) $$Ta_{i}=\frac{To_{i}}{d_{i}}=\frac{\sum_{j=1}^{T}t_{i,j}}{d_{i}}$$

where *t_i,j_* represents the operating time of vehicle *i* on the *j*-th day during the statistical period *T*.

(3) Definition 3: Operating-Days Ratio

The operating-days ratio (*τ*) is defined as the ratio of the number of operating days *d_i_* of vehicle *i* during a statistical period of *T* days to the total statistical period *T*, as shown in Equation (9). The value of *τ* ranges from 0 to 1, with a value closer to 1 indicating that the vehicle operated on more days within the statistical period.

(9) $$\tau =\frac{d_{i}}{T}$$

### 3.2. Calculation of Vehicle Operational Characteristic Indicators

**Figure 1:**
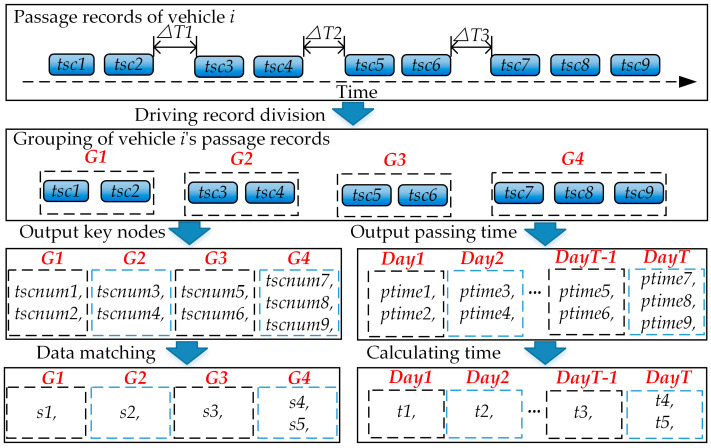
Calculation process of vehicle operational characteristic indicators.

The calculation of identification indicator data for each vehicle within the set of passage records *R* is conducted, following the steps shown in Figure 1.

#### 3.2.1. Construction of Bayonet Distance Matrix

The bayonet distance matrix for adjacent points is constructed based on the latitude and longitude information from urban traffic surveillance attributes. For adjacent traffic surveillance bayonets *tsc_i_* and *tsc_j_* in the city, the shortest path between them is determined based on the urban road network, and the path is divided into several key nodes (*x*_1_, *y*_1_), (*x*_2_, *y*_2_), …, (*x_n_*, *y_n_*). The distance between adjacent nodes is calculated and summed to determine the total distance between adjacent traffic surveillance bayonets *tsc_i_* and *tsc_j_*, as shown in Equation (10).

(10) $$s_{i,j}=\sum_{1}^{i}Re*\mathrm{ar}\cos[\cos(y_{n-1})*\cos(y_{n})*\cos(x_{n-1}-x_{n})+\sin(y_{n-1})*\sin(y_{n})]$$

where *Re* represents the Earth’s radius, which is 6371.0 km.

The distance matrix for m adjacent traffic surveillance bayonets is established as shown in Table 4. where *l_im_* represents the road segment distance between adjacent traffic surveillance bayonet *i* and bayonet *m*, measured in kilometers.

#### 3.2.2. Segmentation of Passage Records

Vehicle passage records are segmented based on the time intervals between adjacent traffic surveillance bayonets [29]. Based on the average time for all vehicles to pass through the bayonets, this study sets the time interval threshold Δ*T* to 15 min. When the time interval for vehicle *j* to pass through adjacent traffic surveillance bayonets *tsc_i_* and *tsc_i+1_* meets the condition *ptime_i+1_* − *ptime_i_* ≥ Δ*T*, the passage records read by the adjacent traffic surveillance bayonets *tsc_i_* and *tsc_i+1_* are segmented into two separate short-term trips. Finally, the set of driving records *R_j_* for vehicle *j* within the statistical period is divided into short-term trips {*G1*, *G2*, …, *Gm*}.

#### 3.2.3. Calculation of Driving Mileage

The key nodes for vehicle *j* in different short-term trips are extracted, matched with the bayonet distance matrix to determine the mileage *s* of each trip, and finally, the total mileage *S* of vehicle *j* is calculated. The steps are as follows:

(1) The traffic-surveillance-bayonet numbers {*tsc1*, *tsc2*, …, *tsck*} for vehicle *j* in the *i*-th trip are extracted as the key nodes of the short-term trip *Gi*, where *k* represents the total number of traffic surveillance bayonets passed during the i-th trip.
(2) The key nodes {*tsc1*, *tsc2*, …, *tsck*} of the short-term trip *Gi* are matched with the bayonet distance matrix to obtain the distances {*l*_1_, *l*_2_, …, *l_k__−_*_1_} between adjacent bayonets. The driving mileage *s_i_* of the short-term trip *Gi* can then be calculated, as shown in Equation (11).

(11) $$s_{i}=\sum_{w=1}^{k-1}l_{w}$$

where *w* represents the distance between the *w*-th pair of adjacent bayonets in the short-term trip *Gi*, measured in kilometers.

(3) The driving mileage *s_i_* for all short-term trips of vehicle *j* within the statistical period *T* is calculated, and the total driving mileage *S_j_* of vehicle *j* during the statistical period is then determined, as shown in Equation (12).

(12) $$S_{j}=\sum_{i=1}^{m}s_{i}$$

#### 3.2.4. Calculation of Driving Time

Similar to Section 3.2.3, the short-term trips *Gi* of vehicle *j* for each day within the statistical period *T* are extracted. The driving time *ts* for each short-term trip of vehicle *j* is then calculated, and finally, the total operating time *To* and the number of days *d_j_* the vehicle was in operation during the statistical period are determined. The steps are as follows:

(1) The number of days *d_j_* within the statistical period during which vehicle *j* has driving records, along with the corresponding short-term trips, is calculated. Days without vehicle passage records are marked as 0.
(2) The passage times {*ptime1*, *ptime2*, …, *ptimek*} at the traffic surveillance bayonets for vehicle *j* during the i-th trip are extracted, and the driving time *ts_i_* for that trip is calculated, as shown in Equation (13).

(13) $$ts_{i}=ptimek-ptime1$$

(3) The driving time for all short-term trips of vehicle *j* within the statistical period *T* is calculated, and the total operating time *To* of vehicle *j* during the statistical period is determined, as shown in Equation (14).

(14) $$To_{j}=\sum_{i=1}^{m}ts_{i}$$

### 3.3. Identification Indicator Determination

**Figure 2:**
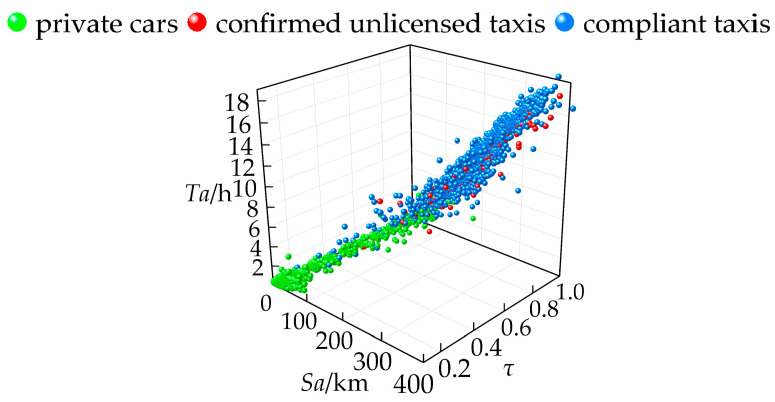
Distribution characteristics of the training sample.

The average daily mileage *Sa*, average daily operating time *Ta*, and operating-days ratio *τ* for private cars, confirmed unlicensed taxis, and compliant taxis in the training sample *υ* were calculated, with the distribution shown in Figure 2. The results show that *Sa*, *Ta*, and *τ* effectively characterize the spatiotemporal differences between private cars, confirmed unlicensed taxis, and compliant taxis. Most private cars are concentrated in segments with low average daily mileage and operating time, but their operating days are widely dispersed. Confirmed unlicensed taxis and compliant taxis exhibit a high degree of similarity, with most having significantly higher average daily mileage, operating time, and operating-days ratio, compared to private cars.

A variance analysis was conducted on the training sample *υ*, comparing private cars, confirmed unlicensed taxis, and compliant taxis across three dimensions. The analysis aimed to explore the extent to which these parameters differentiate the operational characteristics of different vehicle types, with results shown in Table 5. The results show that the average daily mileage for private cars is 21.926 km, while for confirmed unlicensed taxis and compliant taxis it is 175.291 km and 175.058 km, respectively. The average daily operating time for private cars is 0.738 h, while for confirmed unlicensed taxis and compliant taxis it is 8.125 h and 8.762 h, respectively. The operating-days ratio for private cars is 0.561, while for confirmed unlicensed taxis and compliant taxis it is 0.976 and 0.975, respectively. The data distribution for private cars across the three dimensions is significantly lower than that for both confirmed unlicensed taxis and compliant taxis. Therefore, this study uses *Sa*, *Ta*, and *τ* as identification indicators for detecting suspected unlicensed taxis.

Therefore, based on the basic definitions of the identification indicators, the identification indicator data for 252,332 vehicles were calculated. The results are shown in Table 6.

## 4. Identification Method

Binary Logistic Regression is a probabilistic model primarily used for regression analysis when the dependent variable is binary. The independent variables can be either categorical or continuous. Due to its advantages, including an explicit probability expression, fast solution speed, and high prediction accuracy, it is widely used in the field of traffic behavior analysis and prediction [30,31]. Based on Binary Logistic Regression, this study constructs an unlicensed-taxi-identification model using significant identification indicators. The influence of different indicators on the discrimination of unlicensed taxis is converted into a probability range between 0 and 1, enabling the detection and identification of unlicensed taxis.

### 4.1. Suspected-Unlicensed-Taxi-Identification Model

Assume that in the filtered bayonet vehicle-passage information, the vehicle usage type *Y* is a binary variable. When *Y* = 0, it represents a private car; when *Y* = 1, it represents an unlicensed taxi. Then *P* = *P* (*Y* = 1) represents the probability that the vehicle is an unlicensed taxi, and *P*(*Y* = 0) = 1 − *P* represents the probability that the vehicle is a private car. *x_1_*, *x_2_*, …, *x_m_* are *m* factors affecting the vehicle type. According to the binary Logistic model, the probabilities that the vehicle is an unlicensed taxi or a private car are given by Equations (15) and (16), respectively.

(15) $$P=\frac{e^{\beta_{0}+\beta_{1}x_{1}+\beta_{2}x_{2}+\cdots +\beta_{m}x_{m}}}{1+e^{\beta_{0}+\beta_{1}x_{1}+\beta_{2}x_{2}+\cdots +\beta_{m}x_{m}}}$$

(16) $$P(Y=0)=1-P=\frac{1}{1+e^{\beta_{0}+\beta_{1}x_{1}+\beta_{2}x_{2}+\cdots +\beta_{m}x_{m}}}$$

where *β*_0_ is the intercept, and *β*_1_, *β*_2_, …, *β_m_* are the partial regression coefficients.

### 4.2. Parameter Estimation

The parameters in the binary Logistic model are estimated using the Maximum Likelihood Estimation (MLE) method. This process involves first constructing the likelihood function and then determining the parameter estimates that maximize it. First, for the existing sample, the likelihood function is established as shown in Equation (17).

(17) $$L=\prod_{i=1}^{n}P_{i}^{Y_{i}}{\left(1-P_{i}\right)}^{1-Y_{i}}$$

where *i* = 1, 2, …, *n* represents the *i*-th vehicle sample; *L* is the likelihood function, with a range of [0, 1]; *Y_i_* is the value of the binary variable corresponding to vehicle *i*; and *P_i_* is the probability that the vehicle is an unlicensed taxi.

The log-likelihood function of the likelihood function is given in Equation (18). By taking the first derivative of ln*L* setting it to zero, and then applying the Newton–Raphson iterative method to solve the resulting system of equations, the maximum likelihood estimates and standard errors of the parameters can be obtained.

(18) $$\ln L=\sum_{i=1}^{n}\left[Y_{i}\ln P_{i}+(1-Y_{i})\ln(1-P_{i})\right]$$

In this study, IBM SPSS Statistics 26 was used to perform binary Logistic regression modeling based on the training sample *υ* consisting of private cars and unlicensed taxis. The calculation and analysis results of various statistics are shown in Table 7.

The unlicensed-taxi-identification model is obtained as shown in Equation (19), where *P* represents the probability that the vehicle is an unlicensed taxi. When *P* ≥ 0.5, the vehicle is considered a suspected unlicensed taxi. *x*_1_, *x*_2_*,* and *x*_3_ represent the vehicle’s average daily mileage, average daily operating time, and operating-days ratio, respectively.

(19) $$P=\frac{e^{-11.885+0.027x_{1}+1.488x+5.409x_{3}}}{1+e^{-11.885+0.027x_{1}+1.488x+5.409x_{3}}}$$

### 4.3. Model Validation

The Hosmer–Lemeshow test assesses the degree of agreement between the fitted values and the observed values. A significance level greater than 0.05 indicates that the model passes the Hosmer–Lemeshow test. As shown in the Hosmer–Lemeshow test results in Table 8, the significance level is 0.328, which is greater than 0.05, indicating that the binary Logistic model has a good fit.

### 4.4. Model Performance and Predictive Ability Evaluation

The goodness-of-fit evaluation of the model only suggests that the information regarding private cars and unlicensed taxis in the training sample has been adequately captured, which does not necessarily indicate that the model has strong predictive performance. Therefore, an evaluation of the model’s predictive power is also necessary [32]. Therefore, this study employs recall (*R*), precision (*P*), accuracy (*ACC*), and F1 score (*F*1) as performance evaluation metrics, and plots the ROC curve to assess the model’s predictive ability.

The formulas for calculating the model performance metrics based on the confusion matrix are presented in Equations (13)–(16). Specifically, *TP* refers to the number of samples that are actually positive and predicted as positive, i.e., the number of unlicensed taxis correctly identified as unlicensed taxis; *FP* refers to the number of samples that are actually negative but predicted as positive, i.e., the number of private cars incorrectly predicted as unlicensed taxis; *FN* refers to the number of samples that are actually positive but predicted as negative, i.e., the number of unlicensed taxis incorrectly predicted as private cars; and *TN* refers to the number of samples that are actually negative and predicted as negative, i.e., the number of private cars correctly predicted as private cars.

(20) $$R=\frac{TP}{TP+FN}$$

(21) $$P=\frac{TP}{TP+FP}$$

(22) $$ACC=\frac{TP+TN}{TP+TN+FP+FN}$$

(23) $$F1=\frac{2PR}{P+R}$$

The confusion matrix obtained from the experimental results is shown in Table 9. The model’s performance-evaluation metrics are 89.26%, 94.74%, 99.10%, and 91.91%, respectively, indicating that the model can effectively distinguish between private cars and unlicensed taxis. Moreover, the model’s accuracy is 99.10%, which is higher than that of related studies [9,14,16,18,19], further demonstrating the high recognition performance of the unlicensed-taxi-identification model constructed using binary Logistic regression.

**Figure 3:**
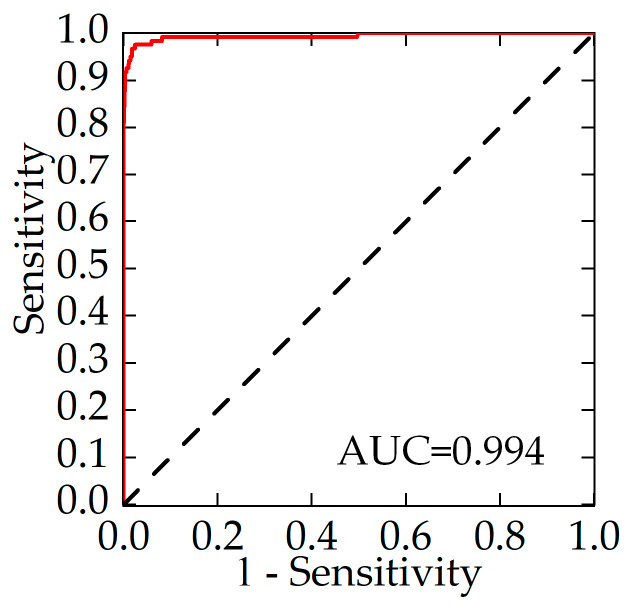
ROC curve of unlicensed-taxi-identification model.

When evaluating the predictive ability of the unlicensed-taxi-identification model using the ROC curve, the greater the curve’s bulge toward the top-left corner and the closer the area under the curve (AUC) value is to 1, the better the model’s predictive performance. The model’s predicted probabilities are used as test variables, and private cars and unlicensed taxis in the training sample are used as status variables for ROC analysis. The results are shown in Figure 3. As shown in the figure, the AUC is 0.994, indicating that the unlicensed-taxi-identification model has a strong predictive capability for vehicle attributes.

## 5. Empirical Analysis

### 5.1. Identification of Suspected Unlicensed Taxis

**Figure 4:**
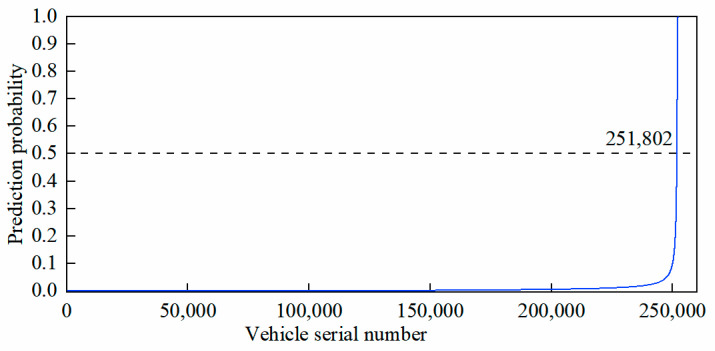
Probability distribution of vehicles engaging in unlicensed-taxi activities.

Based on the unlicensed-taxi-identification model, Figure 4 presents the probability distribution characteristics of 252,332 vehicles engaging in unlicensed-taxi activities. As shown in Figure 4, 251,802 vehicles have a low probability (*P* < 50%) of engaging in unlicensed-taxi activities, while 530 vehicles have a high probability (*P* > 50%) of engaging in such activities.

Vehicles with a high probability of engaging in unlicensed-taxi activities are defined as suspected unlicensed taxis, as shown in Table 10.

### 5.2. Operational Characteristic Analysis

#### 5.2.1. Mileage Characteristics

**Figure 5:**
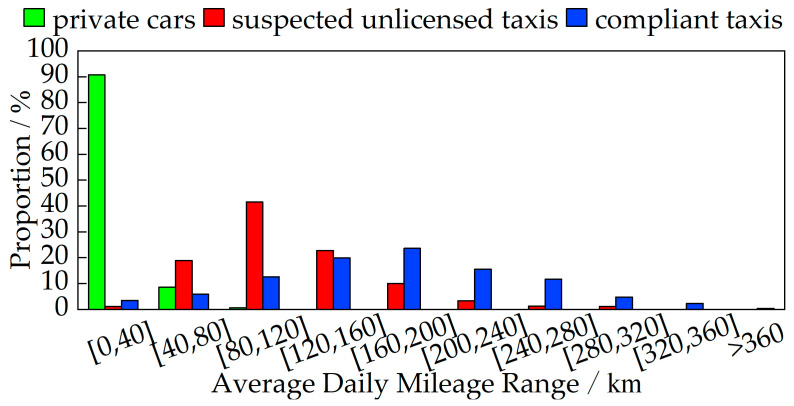
Distribution of average daily mileage for three types of vehicles.

This study analyzes the mileage characteristics among private cars, suspected unlicensed taxis, and compliant taxis. A total of 99.31% of private cars have an average daily mileage of less than 80 km, while 80.00% of suspected unlicensed taxis and 90.55% of compliant taxis have an average daily mileage of more than 80 km. Overall, compliant taxis have the highest average daily mileage, followed by suspected unlicensed taxis, with private cars having the lowest, as shown in Figure 5.

Considering the practical context, this phenomenon can be explained with regard to the following two aspects. (1) Private cars are primarily used for daily commuting and other personal activities, resulting in lower mileage. In contrast, suspected unlicensed taxis and compliant taxis, as commercial vehicles, require long-term operation for passenger transportation, leading to higher mileage. (2) Compared to suspected unlicensed taxis, compliant taxis not only possess legal operating licenses, but also enjoy greater visibility, which results in more ride orders. Therefore, the overall mileage of compliant taxis is higher than that of suspected unlicensed taxis.

#### 5.2.2. Operating-Time Characteristics

To explore the differences in time distribution among different types of vehicles, this study analyzes the average operating days, average daily operating time, and operating periods within each day, for different vehicle types.

**Figure 6:**
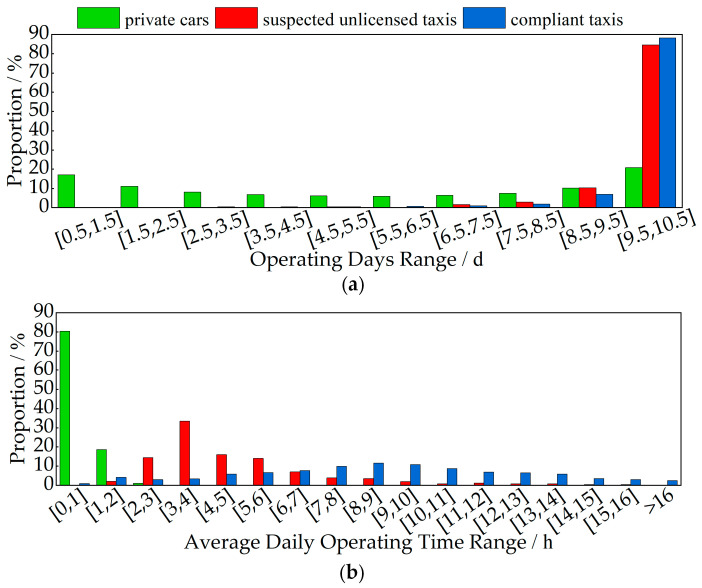
Operating-time-characteristic distribution: (**a**) operating days; (**b**) average daily operating time.

Figure 6a shows the average operating days of different types of vehicles over a 10-day statistical period. As shown in the figure, in the interval where the average operating days are no less than 9 days, suspected unlicensed taxis account for 94.91%, compliant taxis account for 95.25%, and private cars account for 31.04%. Therefore, the average operating days of compliant taxis and suspected unlicensed taxis are similar, both concentrated in the higher range of operating days, while the distribution of operating days for private cars is more even.

Figure 6b shows the average daily operating time of different types of vehicles over a 10-day statistical period. Compared to suspected unlicensed taxis and compliant taxis, private cars have the highest number of vehicles with an average daily operating time of 0 to 2 h, accounting for 98.86% of all private cars, and a maximum operating time of no more than 5 h. The daily operating time of suspected unlicensed taxis mainly ranges from 1 to 10 h, with the majority concentrated in the 3 to 4 h range, accounting for 33.40%. Compliant taxis have a wider range of average daily operating times, ranging from 1 to 15 h.

**Figure 7:**
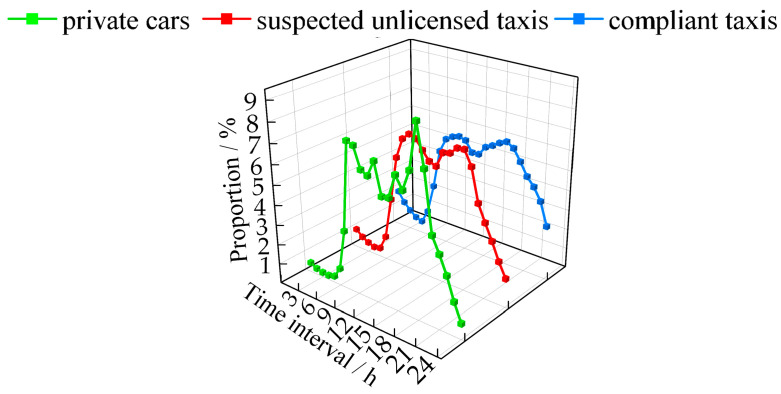
Distribution characteristics of operating time periods within a day.

Figure 7 shows the distribution of daily operating periods for different types of vehicles over a 10-day statistical period. As shown in the figure, the main operating period for private cars and suspected unlicensed taxis is from 7:00 to 21:00 each day, while the main operating period for compliant taxis is from 7:00 to 24:00. During the secondary operating periods (0:00 to 7:00 and 21:00 to 24:00) for private cars and suspected unlicensed taxis, the distribution proportion of suspected unlicensed taxis and compliant taxis is higher than that of private cars. Additionally, the distribution of all three types of vehicles during the morning and evening peak hours (7:00 to 9:00 and 17:00 to 20:00) is higher than at other times, with private cars showing the most significant peak.

In summary, in terms of operating time, suspected unlicensed taxis exhibit characteristics such as a higher number of operating days, longer daily operating times, and a higher proportion of night-time operation, which are similar to those of compliant taxis. In contrast, private cars display lower levels of these characteristics. This is consistent with real-world situations: private cars typically have shorter daily driving times and mainly operate during the day, whereas both compliant taxis and unlicensed taxis need to operate continuously throughout the day and night to transport passengers and earn higher income.

### 5.3. Spatiotemporal-Distribution Characteristics Analysis

#### 5.3.1. Overall Spatial-Distribution Characteristics

**Figure 8:**
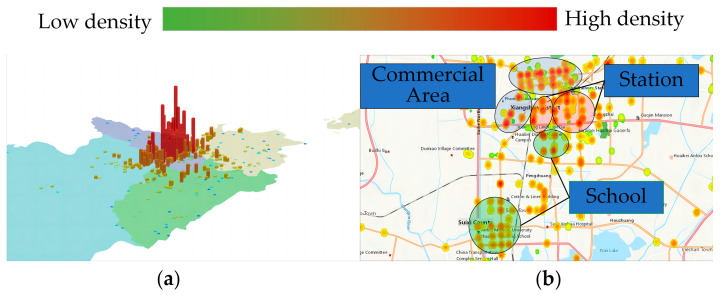
Main operating areas of suspected unlicensed taxis: (**a**) overall distribution; (**b**) operating hotspot areas.

The main operating areas of suspected unlicensed taxis in this city are shown in Figure 8. Figure 8a shows the overall distribution of operating areas for suspected unlicensed taxis in this City. They are mainly concentrated in the main urban area, with the highest density in the central urban area. Figure 8b further analyzes the distribution characteristics of vehicle operations within the main urban area. As shown by the circled regions in the figure, suspected unlicensed taxis are primarily distributed around stations, universities, parks, and commercial areas within the main urban area. This distribution aligns with the common destinations of passengers, suggesting that management authorities should intensify inspections in these areas.

#### 5.3.2. Temporal- and Spatial-Distribution Characteristics of Travel Origins

This study selects a suspected unlicensed taxi and records its first- and last-passage information daily at traffic surveillance bayonets, to examine the temporal and spatial distribution characteristics of the taxi’s travel origins.

**Figure 9:**
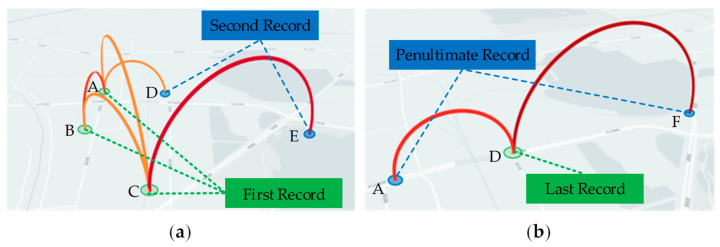
Distribution of traffic surveillance bayonets passed by the suspected unlicensed taxi each day: (**a**) first pass; (**b**) last pass.

During the statistical period, the distribution of traffic surveillance bayonets where the suspected unlicensed taxi passes first and last each day is shown in Figure 9. It can be observed that the first bayonet the taxi passes each day is not unique, but rather, one of three closely spaced bayonets: A, B, or C. However, the last bayonet the taxi passes each day is fixed, and is always bayonet D. Based on the layout of the city’s traffic surveillance bayonets, it can be inferred that bayonets A, B, C, and D are positioned at closely spaced intersections along adjacent road segments. The suspected unlicensed taxi’s daily travel origin and destination points show a high degree of overlap, indicating that this location is likely the driver’s residence. Management authorities can leverage this information, along with the driver’s daily routine, to implement targeted regulation.

**Figure 10:**
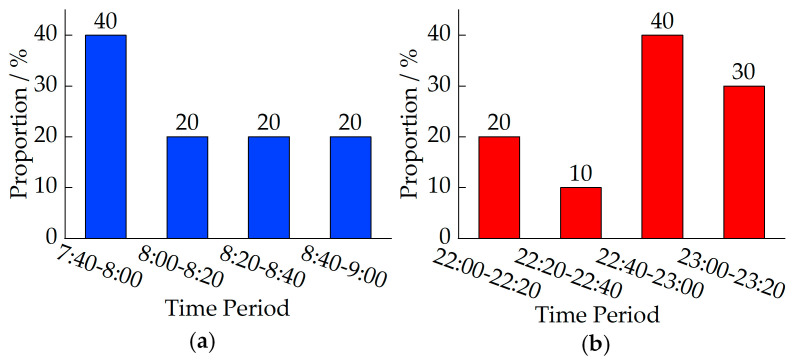
Temporal distribution of traffic surveillance bayonets passed by the suspected unlicensed taxi during the statistical period: (**a**) start time; (**b**) end time.

Figure 10 shows the temporal distribution of traffic surveillance bayonets where the suspected unlicensed taxi has its first and last passes during the statistical period. As shown in the figure, this suspected unlicensed taxi operated every day during the statistical period, with a wide range of start and end times each day. Additionally, this suspected unlicensed taxi shows a clear concentration in both start and end times, with start times concentrated between 7:40 and 8:00, and end times concentrated between 22:40 and 23:00.

In summary, a targeted enforcement plan can be developed, based on the spatiotemporal-distribution characteristics of suspected unlicensed taxis, to verify whether they are engaged in illegal operations and to ensure precise enforcement.

On the one hand, based on the temporal- and spatial-distribution characteristics of travel origins, traffic management authorities should identify the periods corresponding to the first and last passages of suspected unlicensed taxis through traffic surveillance bayonets, daily. Inspection checkpoints should be preemptively established at intersections or residential areas likely to be traversed by these vehicles. Since the data on suspected unlicensed taxis include vehicle license-plate information, which can be used to retrieve details such as vehicle type and driver identity, precise monitoring can be conducted by comparing whether passing vehicles correspond to the data of the suspected unlicensed taxis. On the other hand, traffic management authorities can utilize the location and time data from the end of the passage record. Based on this, they can retrieve nearby video surveillance footage to verify whether the suspected unlicensed taxi was involved in passenger transport, thereby determining whether it is truly engaged in illegal operations.

The use of the above method enables precise identification and regulation of suspected unlicensed taxis, effectively improving law enforcement efficiency and reducing the costs associated with identifying and managing unlicensed taxis.

## 6. Conclusions

This study employs traffic surveillance bayonets to collect vehicle passage information and analyzes the spatiotemporal characteristics of private cars, confirmed unlicensed taxis, and compliant passenger vehicles. Based on this analysis, identification indicators are established, and vehicle-operation-characteristic metrics are calculated using the aforementioned method. An unlicensed-taxi-identification model is then applied to obtain data on suspected unlicensed taxis, enabling traffic management authorities to develop targeted regulatory strategies for these vehicles. The main conclusions of the study are as follows:

(1) Based on traffic-surveillance-bayonet data, a distance matrix was constructed and a driving interval threshold was set. The mileage and operating time data were calculated, and variance analysis was conducted to compare the differences between private cars, unlicensed taxis, and compliant taxis, leading to the determination of unlicensed-taxi-identification indicators.
(2) Based on the identified unlicensed-taxi indicators, a binary Logistic regression model was established. The model parameters were estimated using the maximum likelihood method, and the model’s goodness-of-fit and predictive power were evaluated through Hosmer–Lemeshow tests and ROC curve analysis. The results show that the model can effectively predict the likelihood of a vehicle engaging in unlicensed-taxi activities (*R* = 89.26%, *P* = 94.74%, *ACC* = 99.10%, *F*1 = 91.91%, AUC = 0.994).
(3) Using the information from the identified suspected unlicensed taxis, an analysis of their daily start and end times and location distribution was conducted to provide a basis for precise management by traffic authorities. The results show that the operational characteristics of suspected unlicensed taxis differ from those of private cars, with regular patterns in their daily start and end times and location distributions.

The study provides precise data support for traffic management authorities, enhancing law enforcement efficiency and contributing to improved passage safety. Furthermore, the study offers a foundation for formulating more rational traffic management policies and promoting the standardization and sustainable development of the transport industry. However, there is still room for further development. Future research will build upon existing studies and the results from traffic management authorities to explore the characteristics of groups involved in illegal operations. This will inform the development of a preventive regulatory identification method for unlicensed taxis, facilitating proactive supervision during processes such as new vehicle purchases and second-hand vehicle transactions.

## 7. Patents

The work related in this study has obtained a Chinese patent, grant number CN202111474233.1.

## Figures and Tables

**Table 1:** Types and meanings of traffic-surveillance-bayonet vehicle passage data.

Number	Data Type	Example	Meaning
1	*pnum*	1832926172	The count of vehicles passing through traffic surveillance bayonet
2	*vnum*	Wan F****1	Vehicle license plate number
3	*vncol*	Blue	Vehicle license plate color
4	*tscnum*	34060300001190110040	Traffic surveillance bayonet number
5	*ptime*	2021/12/1 6:59:05	The time the vehicle passes through the traffic surveillance bayonet
6	*pd*	9.0	Vehicle driving direction
7	*pspeed*	46	The speed of the vehicle passing through the traffic surveillance bayonet

**Table 2:** Types and meanings of traffic-surveillance-bayonet location information data.

Number	Data Type	Example	Meaning
1	*tscnum*	340621000011901401	Traffic-surveillance-bayonet number
2	*lon*	116.764	Longitude of the traffic surveillance bayonet
3	*lat*	33.922	Latitude of the traffic surveillance bayonet

**Table 3:** Type of driving data error.

Error Identification Information	Cause of Error
05377	Incomplete License Plate Information
0B70641	Misidentification of Leading “0” in Number

**Table 4:** Adjacent-traffic-surveillance distance matrix.

Serial Number	*tsc1*	*tsc2*	*…*	*tsc_i_*	*…*	*tsc_m_*
*tsc1*	*0*	*l*_12_	*…*	*l*_1*i*_	*…*	*l*_1*m*_
*tsc2*	*l*_21_	*0*	*…*	*l*_2*i*_	*…*	*l*_2*m*_
*…*	*…*	*…*	*…*	*…*	*…*	*…*
*tsc* ***_i_***	*l_i_*_1_	*l_i_*_2_	*…*	*0*	*…*	*l_im_*
*…*	*…*	*…*	*…*	*…*	*…*	*…*
*tsc* ***_m_***	*l_m_*_1_	*l_m_*_2_	*…*	*l_m_*_3_	*…*	*0*

**Table 5:** Variance analysis of identification indicators.

		Mean	Standard Deviation	F	Sig	Post Hoc Test
*Sa*	private cars	21.926	14.571	4328.400	0.00	I < II; I < III
	confirmed unlicensed taxis	175.291	79.383			
	compliant taxis	175.058	71.649			
*Ta*	private cars	0.738	0.426	4404.217	0.00	I < II; I < III
	confirmed unlicensed taxis	8.125	3.875			
	compliant taxis	8.762	3.776			
*τ*	private cars	0.561	0.342	1341.889	0.00	I < II; I < III
	confirmed unlicensed taxis	0.976	0.078			
	compliant taxis	0.975	0.094			

Note: In the post hoc test, I represents private cars, II represents unlicensed taxis, and III represents compliant taxis.

**Table 6:** Filtered vehicle-identification-indicator data.

*vnum*	*Sa*/km	*Ta*/h	*τ*
Wan F****9	310.027	12.464	1
Wan F****2	302.631	15.682	1
…	…	…	…
Wan L****5	1.000	0.410	0.8

**Table 7:** Maximum-likelihood estimation results.

Variables.	B	S.E	Wald	df	Sig	Exp (B)
*Sa*	0.027	0.014	3.936	1	0.047	1.027
*Ta*	1.488	0.361	16.997	1	0.000	4.430
*τ*	5.409	1.916	7.973	1	0.005	223.487
Constant	−11.885	1.916	38.461	1	0.000	0.000

**Table 8:** Hosmer–Lemeshow test.

Steps	Chi-Square	df	Sig
1	9.175	8	0.328

**Table 9:** Confusion matrix results.

Category		Predicted Values	
		Unlicensed Taxis	Private Cars
Actual values	Unlicensed taxis	108	13
	Private cars	6	1994

**Table 10:** Information on suspected unlicensed taxis.

*vnum*	*Sa*/km	*Ta*/h	τ
Wan F****2	302.631	15.682	1
Wan F****0	297.365	15.492	1
…	…	…	…
Wan F****2	76.443	2.956	1

## Data Availability

The datasets used or analyzed during the current study are available from the corresponding author on reasonable request.
